# How Discoloration of Porcine Cruor Hydrolysate Allowed the Identification of New Antifungal Peptides

**DOI:** 10.3390/foods11244035

**Published:** 2022-12-14

**Authors:** Aurore Cournoyer, Jacinthe Thibodeau, Laila Ben Said, Zain Sanchez-Reinoso, Sergey Mikhaylin, Ismail Fliss, Laurent Bazinet

**Affiliations:** 1 Department of Food Science, Université Laval, Québec, QC G1V 0A6, Canada; 2 Laboratoire de Transformation Alimentaire et Procédés ÉlectroMembranaires (LTAPEM, Laboratory of Food Processing and Electromembrane Process), Université Laval, Québec, QC G1V 0A6, Canada; 3 Institute of Nutrition and Functional Foods (INAF), Université Laval, Québec, QC G1V 0A6, Canada

**Keywords:** porcine blood, hemoglobin, pepsin, antimicrobials, antifungals, circular economy

## Abstract

Porcine blood is an important by-product from slaughterhouses and an abundant source of proteins. Indeed, cruor, the solid part of blood, is mainly composed of hemoglobin. Its enzymatic hydrolysis with pepsin generates a diversity of peptides, particularly antimicrobials. One of the downsides of using these hydrolysates as food bio-preservatives is the color brought by the heme, which can be removed by discoloration. Nonetheless, the effects of this procedure on the antimicrobial peptide population have not been completely investigated. In this study, its impacts were evaluated on the final antibacterial and antifungal activities of a cruor hydrolysate. The results demonstrated that 38 identified and characterized peptides showed a partial or total decrease in the hydrolysate, after discoloration. Antifungal activities were observed for the raw and discolored hydrolysates: MICs vary between 0.1 and 30.0 mg/mL of proteins, and significant differences were detected between both hydrolysates for the strains *S. boulardii*, *C. guilliermondii*, *K. marxianus*, *M. racemosus* and *P. chrysogenum*. The raw hydrolysate showed up to 12 times higher antifungal activities. Hence, peptides with the highest relative abundance decrease after discoloration were synthesized and tested individually. In total, eight new antifungal peptides were characterized as active and promising. To our knowledge, this is the first time that effective antifungal peptide sequences have been reported from porcine cruor hydrolysates.

## 1. Introduction

The Canadian porcine industry is the third largest pork exporter in the world, which represents about 70% of the Canadian pork production [1]. Moreover, about 21.7 kg of pork are consumed per year by Canadians, making it also a staple of the Canadian diet [2]. However, only 60 to 62% of a pig is dedicated to human consumption, while the rest is considered as undervalued by-products [3]. Slaughterhouses release by-products such as skin, bones, inedible offal, and blood. Among them, blood represents about 21 million liters in Quebec per year, and approximatively 68 million liters per year in Canada [4]. The use of blood is not legislated and depends on each company’s investment for the management of waste such as the quality and safety of its recovery. The blood may be used for human or animal consumption, sold to rendering companies, used for industrial composting, incinerated, spread on landfill sites, or buried [5]. Another possible way to valorize this by-product is the production of bio-preservatives that could extend meat shelf life under refrigeration [6]. Indeed, consumers have increasing concerns regarding environmental issues and the use of chemical preservatives to extend meat products’ shelf life [7]. Raw meats are highly perishable products, with a short shelf life. Meat microbial ecosystem includes a large variety of microorganisms that might contribute to the spoilage of products even under refrigerated storage conditions. To overcome this, preservation methods are combined using additional barriers such as ionizing radiation, chemical preservatives, and active packaging [8]. However, with a view to sustainable development and “clean label”, the development of bio-preservation approaches is currently the focus of many studies [9]. Thus, the porcine blood valorization, by producing antimicrobial peptides, could be the start of a new circular model of production. Indeed, the circular economy is an economy that operates in a loop, by preventing the notion of waste. Its main purpose consists of producing goods while strongly limiting the consumption of raw materials and non-renewable energy sources [10].

Blood can be separated in two parts, the plasma, the colorless part, and the cruor, the red cells’ part. Cruor represents 40% of the blood and contains mainly proteins with around 90% hemoglobin. Thanks to its abundance in protein, cruor is an ideal substrate for proteolysis [11,12]. It was demonstrated that the peptic hydrolysis of porcine cruor produces bioactive peptides, but there is still a lack of data concerning them [13,14]. Indeed, most of the research studies on bioactive peptides from hemoglobin were carried out on bovine hemoglobin. Many bioactivities such as antioxidant, antimicrobial, anti-hypertensive, or opioid were reported in the literature on bovine hemoglobin and cruor [15,16,17]. The pioneering identification of antimicrobial peptides from bovine hemoglobin was conducted from the gut content of a tick *Boophilus microplus* [18]. Recently, an in silico comparison between peptides from bovine and porcine hemoglobin demonstrated that their close structures allowed to obtain common bioactive peptides between these two sources [13]. In fact, the α and β chains hemoglobin from bovine and porcine are both similar at 87% and 83%, respectively. Some peptides from porcine cruor hydrolysate have demonstrated antibacterial activities: TSKYR, KLLSHSL and KLLSHSLL, and eight others were highlighted as antibacterial but not tested in a pure form [13]. Moreover, most of these studies have focused on the study of antibacterial activity while very few studies focused on antifungal activity of peptides obtained from bovine hemoglobin [10]. Furthermore, antifungal activity of a porcine cruor hydrolysate has never been reported in the literature.

When an antimicrobial porcine cruor hydrolysate is used directly on meat products, problems regarding the color and metallic flavor produced by the heme might occur. Indeed, the dark brownish color that hemoglobin confers to food formulations restricts its use as an ingredient in meat products, such as light-colored deli meat [19]. Therefore, it is relevant to investigate bioactivities on discolored porcine cruor hydrolysates [20]. The discoloration consists of precipitating the heme at a lowered pH and separating the two phases using centrifugation [21]. This step generates new blood processing operation and by-product, which disagree with the circular economy context of the whole process. Considering those benefits and disadvantages, a raw and discolored cruor hydrolysate were considered and investigated. 

In this context, our study aimed to evaluate the impact of discoloration of porcine cruor hydrolysate on its resulting antimicrobial activities. The specific objectives of the present study were (1) to explore the peptide profiles of the raw and discolored porcine cruor hydrolysates, (2) to identify and characterize the peptides lost by discoloration, (3) to evaluate the antimicrobial (antibacterial and antifungal) activities of both hydrolysates and (4) to synthesize and test new potential antimicrobial peptides.

## 2. Materials and Methods

### 2.1. Porcine Cruor Preparation 

The cruor was directly obtained from raw porcine blood collected from Olymel’s slaughterhouse (Saint-Hyacinthe, QC, Canada). An anticoagulant: Ethylenediaminetetraacetic acid (EDTA at 1 g/L, Sigma-Aldrich, Oakville, ON, Canada), was added to fresh blood to prevent clotting. The red blood cell part was extracted using centrifugations carried out at 13,200× *g* (Avanti J-E Centrifuge, Beckman Coulter, Brea, CA, USA) during 15 min, at 4 °C. The first centrifugation aimed at removing the supernatant, the plasma, and then three subsequent centrifugations were performed to wash the precipitate with sodium chloride (0.9% *w*/*v*, Fisher BioReagents, Waltham, MA, USA). The final precipitate, the cruor, was freeze-dried and stored at −20 °C until its use for enzymatic hydrolysis experiments.

### 2.2. Enzymatic Hydrolysis of Porcine Cruor

#### 2.2.1. Quantification of Hemoglobin Concentration

The hemoglobin concentration in the porcine cruor powder was determined by Drabkin’s method, a quantitative and colorimetric method [22]. The quantification was carried out on a stock solution of 15 g of freeze-dried cruor powder in 100 mL of Milli-Q water, using the Drabkin’s reagent (Sigma-Aldrich, Oakville, ON, Canada). From the determined concentration of the stock solution, the cruor was diluted in Milli-Q water to obtain a precise concentration solution of 2% hemoglobin (*w*/*v*). 

#### 2.2.2. Peptic Hydrolysis Process

Hydrolysis was performed on 3 L of cruor solution prepared in Milli-Q water at a concentration of 2% (*w*/*v*) hemoglobin. Hydrolysis was performed in three separate repetitions. Cruor was digested with pepsin (Sigma-Aldrich, Saint-Louis, MO, USA) from porcine gastric mucosa. The pH was adjusted to 3 with hydrochloric acid (1N, Anachemia, VWR Company International, Lachine, ON, Canada) during 15 min to denature the hemoglobin and reach the reactor pH [13,23,24]. Then, the enzyme (3200–4500 units/mg of protein) was added at an enzyme/substrate ratio of 1/11 (mol/mol). The pH (pH 3) and temperature (23 °C) were maintained constant during the 30 min of hydrolysis. Samples were collected at 0, 2.5, 10 and 30 min for further analyses. The hydrolysis was stopped by increasing the pH to 10 [17] with sodium hydroxide (5 M, Fisher BioReagents, Waltham, MA, USA). The final hydrolysate was freeze-dried and stored at −20 °C.

### 2.3. Discoloration of Porcine Cruor Hydrolysate

The acidification pH for discoloration was selected by testing different pH values: 3.0, 4.0, 4.5, 4.7, 5.0, 5.5 and 6.0 on the final hydrolysate. Then, the absorbance of heme was measured at 392 nm and the pH with the lowest absorbance was chosen. Following these results, pH 4.5 was chosen to discolor a 50 mL sample from each repetition by acidification with HCl 2 N to precipitate the heme. In comparison, optimal pH for the discoloration of bovine hemoglobin was determined at pH 4.7 [25]. After 24 h of decantation at 4 °C, the two phases were separated by centrifugation for 30 min at 6000× *g* (5804R-2, Eppendorf, Hamburg, Germany) [21]. The supernatant consisted of the discolored hydrolysate. The supernatant and the pellet were freeze-dried and stored at −20 °C.

### 2.4. Analyses

#### 2.4.1. Determination of the Degree of Hydrolysis

To quantify the degree of hydrolysis (DH), which corresponds to the number of cleaved peptide bonds depending on the hydrolysis duration, the ortho-phthaldialdehyde (OPA) method was used [26]. The OPA (Sigma-Aldrich, Oakville, ON, Canada) reagent solution was made from Sodium Dodecyl Sulfate 20% (*w*/*v*) (SDS, Fisher Scientific, Fair Lawn, NJ, USA), Sodium tetraborate 100 mM (Fisher Chemicals, Merelbeke, Belgium), solid OPA diluted in methanol at 4% (*w*/*v*) (Fisher Chemicals, Belgium) and β-mercaptoethanol 0.2% (*v*/*v*) (Sigma-Aldrich, Oakville, ON, Canada). The calibration curve was made using D-L-leucine (Sigma-Aldrich, Oakville, ON, Canada) diluted with SDS 1% (*w*/*v*). Standards and diluted samples (1:15) were in contact with the reagent for exactly 2 min, at room temperature, to complex with α-amino groups released during hydrolysis. The proportion of cleaved bonds was quantified by spectrophotometry at 340 nm (HP8453, Agilent Technologies, Santa Clara, CA, USA) and compared with the total cleavable bonds for hemoglobin, 8.3 meq/g of protein [27,28]. 

#### 2.4.2. Protein Content

To determine the protein content in the hydrolysates after the enzymatic hydrolysis and the discoloration, the Dumas method was performed using a rapid MicroNcube (Elementar, Langenselbold, Germany), on freeze-dried samples. The total nitrogen in the sample was measured and the protein concentration was estimated with a conversion factor of 6.25 [27].

#### 2.4.3. RP-UPLC-MS/MS Peptide Profiles Analysis

RP-UPLC coupled with MS/MS Q-TOF system was used to separate, identify, and characterize the peptide population, present in the porcine cruor hydrolysates. RP-UPLC tests were operated using a 1290 Infinity II UPLC (Agilent Technologies, Santa Clara, CA, USA), following the method proposed by [10]. This chromatography instrument was composed of a binary pump (G7120A), a multisampler (G7167B), an in-line degasser and a variable wavelength detector (VWD G7114B) adjusted to 214 nm. The mobile phase composition followed a gradient elution consisting of solvent A (LC-MS grade water with 0.1% formic acid) and solvent B (LC-MS grade acetonitrile with 0.1% formic acid) applied, at constant temperature of 23 °C, with solvent B going from 1% to 13% in 6 min, increasing to 35% until 25 min. Then, solvent B ramped to 100% until 35 min and was maintained for 10 min before returning to initial conditions. The cruor hydrolysate samples (2% protein (*w*/*v*)) were filtered through 0.22 µm PVDF filter and a volume of 0.25 µL was loaded onto a Poroshell 120 EC-C18 column (2.1 × 100 mm i.d., 2.7 micron, Agilent, Santa Clara, CA, USA) at a flow rate of 500 µL/min. 

The determination of the accurate mass was performed using a hybrid ion mobility quadrupole time of flight mass spectrometer (6560 high-definition mass spectrometry (IM-Q-TOF), Agilent, Santa Clara, CA, USA). Calibration was completed using an ESI-L low concentration tuning mix (G1969-85000, Agilent Technologies, Santa Clara, CA, USA). Nitrogen was selected as the drying (13.0 L/min, 150 °C) and nebulizer gas (30 psi). The voltages were set at 3500 V for the capillary, 300 V for the nozzle and 400 V for the fragmentor. Positive mode at Extended Dynamic Range, 2 Ghz, 3200 *m/z* was used to record signals. Data acquisition and analysis were performed using the Agilent Mass Hunter Software package (LC/MS Data Acquisition, Version B.08.00 and Qualitative Analysis for IM-MS, Version B.07.00 Service Pack 2 with BioConfirm Software and BioConfirm, Version 10.0). A false discovery rate of 1% was used for extraction of biomolecules. Then, a matching was performed with the known sequences of the α and β chains of the porcine hemoglobin (UniProtKB—P01965, P02101) to obtain all the peptide sequences found in the hydrolysates. Finally, these peptides were compared with bioactive peptides already known in the literature or analyzed with a predictive antimicrobial database [29].

### 2.5. Synthesized Peptides 

The peptide synthesis was performed by Laboratoire Innodal (Lévis, QC, Canada). Fourteen peptides were synthesized by standard solid-phase peptide synthesis (SPPS) on an automated peptide synthesizer [30]. Solubility of peptides was first tested on saline water with 0.85% NaCl. Finally, as most synthesized peptides had a high hydrophobicity, an organic solvent was used. Each peptide was solubilized in a small volume of dimethylsulfoxide (DMSO), and water was subsequently added until a 10% (*v*/*v*) DMSO concentration was reached [31]. The stock solution of each peptide was at a concentration of 2.5 mM. The pH of the final solution was below the isoelectric point of each peptide. Thus, all peptides were positively charged. A negative control of the solution of DMSO 10% (*v*/*v*) was used to ensure the results were not affected by the solvent. Antifungal assays on synthesized peptides were realized using methods described below. 

### 2.6. Antimicrobial Assays

#### 2.6.1. Microorganisms

The antibacterial activity was tested against two indicator bacteria including *Escherichia coli* and *Listeria ivanovii* (Table 1). These two strains are Gram-negative and Gram-positive, respectively and are commonly sensitive strains. They were subcultured in Tryptic Soy Broth (TSB) two times for 18 h, at 37 °C for *E. coli* and 30 °C for *L. ivanovii*. The antifungal activity was evaluated against four filamentous molds and four yeasts (Table 1). All the strains for antifungal assays were chosen since they are the most often strains identified as spoilage microorganisms of food products, specifically in the meat industry [32]. The 4 filamentous molds were subcultured on Potato Dextrose Agar (PDA) at 25 °C, for 7 days, 2 times. The 4 yeasts were subcultured in Potato Dextrose Broth (PDB) at 25 °C, for 48 h, 2 to 3 times (Table 1).

#### 2.6.2. Agar Diffusion Assay

An agar diffusion method was realized to observe the growth inhibition of hydrolysates or peptides [10]. The antibacterial activity of the hydrolysate was tested on Tryptic Soy Agar (TSA, BD-Difco, Sparks, MD, USA), and antifungal activity was evaluated on PDA. To prepare the bacteria inoculum, the pre-culture was diluted at 10^6^ CFU/mL. For the conidia from the molds, they were recovered after pre-culture with a swab, soaked with a solution of peptone water (10% *w*/*v*) with Tween 80 (0.1% *w*/*v*). The conidia were added to 5 mL of the same solution. The yeasts were directly enumerated from the 48 h culture diluted (1:10). The cells concentration was determined in a solution of methylene blue, by using an automated cell counter (Invitrogen™ AMQAF1000, Oakville, ON, Canada). Then, the inoculum was adjusted to 10^6^ CFU/mL. Once the media of TSA or PDA reached 45 °C, it was inoculated at 1% (*v*/*v*) with the inoculum of the tested microorganism and poured in a Petri dishes. Wells were made in the solidified agar to receive 80 µL of the samples. Hydrolysate samples were tested at a concentration of 40 mg/mL of proteins. The incubation was of 24 h at 37 °C and 30 °C, respectively for *E. coli* and *L. ivanovii*, 48–72 h at 25 °C for molds and *R. mucilaginosa*, and finally at 35 °C for the three other yeasts. For the assays with porcine cruor hydrolysates, reuterin (192 mM) was used as a positive control, bacteriocin generally active against bacteria and fungi [32]. For the assays with the synthesized peptides, natamycin (16.7 µg/mL) (Sigma-Aldrich, Saint-Quentin Fallavier, France) was used as a positive control. The negative control was sterile distilled water. Finally, diameters of the inhibition halos were measured using a ruler (mm), as indicated by the Clinical and Laboratory Standards Institute (CLSI 2016/2017). Tests were realized in triplicate.

#### 2.6.3. Determination of the Minimal Inhibitory, Bactericidal, and Fungicidal Concentrations

The minimum inhibitory concentration (MIC) of the hydrolysate that induces inhibition of the microorganism tested was determined according to Froidevaux et al. [33]. To quantify the growth inhibition, 96-well microplate assays were performed using Mueller-Hinton broth (MH, BD-Difco, Sparks, MD, USA). Samples were micro-diluted from the stock concentration of 40 mg/mL of proteins. The inoculum was pre-diluted in MH at a concentration of 5 × 10^5^ CFU/mL. Then, the microplate was incubated 24 h at 37 °C for *E. coli* and at 30 °C for *L. ivanovii*. The molds and yeasts strains were, respectively tested at 2 × 10^4^ and 1 × 10^5^ conidia or yeast per mL [32]. The automated cell counter was used to determine the concentration. The microplates were incubated for 48 h at 25 °C for the molds and *R. mucilaginosa*, and at 35 °C for the other yeast strains. The MIC was the lowest concentration that inhibited completely the microorganism growth. The growth was measured by absorbance at 595 nm (Infinite^®^ F200 PRO, Tecan Inc., Durham, NC, USA). 

The minimum bactericidal concentration (MBC) or Minimum Fungicidal Concentration (MFC) corresponds to the lowest concentration at which 99.9% of the final inoculum is killed [34]. A volume of 10 µL from MIC determination microplates wells’ showing complete inhibition of the growth were inoculated on the surface of TSA for bacteria and Dichloran Rose-Bengal Chloramphenicol agar (DRBC, BD-Difco, Sparks, MD, USA) plates for molds and yeasts. Then, they were, respectively incubated for 24 h at 37 °C or 30 °C and 48 h at 25 °C. The lowest concentration that inhibited microorganism growth is the MBC or MFC. The ratio MBC/MIC or MFC/MIC allows to determine if the sample is bactericidal/fungicide (≤4) or bacteriostatic/ fungistatic (>4) [32,34]. 

#### 2.6.4. Fractional Inhibitory Concentration Index (FICI)

Microdilution checkerboard assay was performed for determination of the fractional inhibitory concentration (FIC) index, adapted from Hsieh et al. (1993) [35]. Indeed, the purpose of this test was to detect if a synergy occurs between the antifungal peptides highlighted. The highest concentration in the checkerboard was set at 2 times the MIC of the studied peptides. These tests were performed on the most sensitive strains of mold and yeast, *Paecilomyces* spp. and *R. mucilaginosa*. Then, a FIC index was calculated by summing FIC of each peptide from the tested pair. Each FIC was calculated by the ratio of the MIC of synergy and the known MIC of the peptide. Finally, if the FIC index was lower than 0.5, the pair was synergistic, and if it was between 0.5 and 4, the pair was indifferent.

### 2.7. Statistical Analyses

All analyses were completed in triplicates and three independent repetitions of hydrolysis were performed. The results were expressed as mean ± standard deviation. The two types of hydrolysates: raw and discolored, and the pellet of discoloration were compared using an analysis of variance (*p* < 0.05) and Tukey multiple range tests applied with SAS software version 9.4 (for Windows) (SAS institute Inc., Cary, NC, USA). Concerning the microbial results, the MIC et MFC were expressed as mean ± standard deviation. They were compared using a non-parametric alternative for independent data, Kruskal–Wallis one way analysis of variance on ranks, as the data were not continuous. It was followed by a multiple test comparison: Tukey test (*p* < 0.05), applied using SigmaPlot Version 12.0 (Systat Software, Inc., San Jose, CA, USA).

## 3. Results and Discussion

### 3.1. Porcine Cruor Hydrolysis

#### 3.1.1. Degree of Hydrolysis

**Figure 1:**
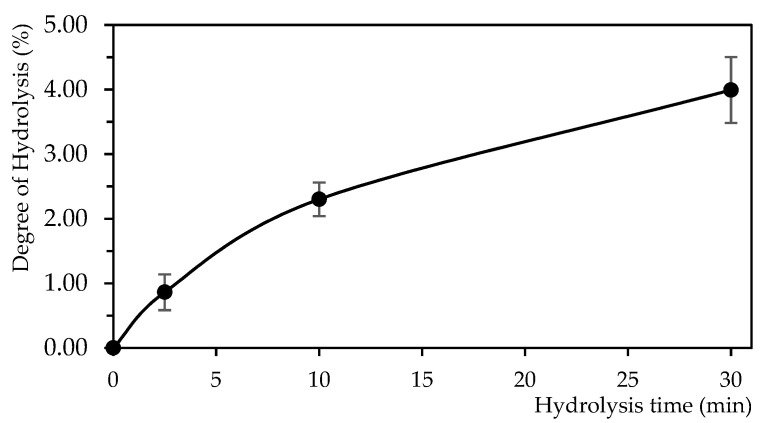
Evolution of the degree of hydrolysis during the 30 min peptic hydrolysis of porcine cruor.

During the hydrolysis of porcine cruor, samples at 0, 2.5, 10 min and final time, were collected to determine the degree of hydrolysis (DH). The DH values increased in a non-linear trend during the 30 min of hydrolysis. The final DH obtained was 4.0 ± 0.5% (Figure 1). The non-linear evolution corroborates the literature on porcine cruor hydrolysate [13]. The DH obtained after 30 min was as reported in the literature for porcine hemoglobin hydrolysed at pH 3 [24] and porcine cruor at pH 2 [36]. In both studies, the DH was below 5% and no plateau was achieved. Indeed, at its optimal conditions (37 °C and pH 2), the pepsin mechanisms of porcine hemoglobin achieve a plateau after 180 min of hydrolysis [24]. To ease the industrial scale-up of the process and reduce the energy consumption due to the heating of the process in the present study, the hydrolysis was conducted at room temperature (23 °C). Moreover, it was shown in several studies that this parameter allowed the production of interesting active peptide populations [6,21,25]. No impact on the hydrolysis efficiency under 30 min of reaction was noted. Samples at 2.5 and 10 min allowed to illustrate how rapidly the porcine hemoglobin was hydrolysate to intermediate peptides. The results confirmed that the denaturation of the protein was complete, and the “zipper” mechanism occurred [37,38]. It was then corroborated by UPLC-MS/MS profiles. The results also confirmed that hemoglobin chains and/or peptides remained and could go through longer peptic hydrolysis. A longer hydrolysis implies a higher DH and generates peptides with lower molecular masses.

#### 3.1.2. Protein Content

**Figure 2:**
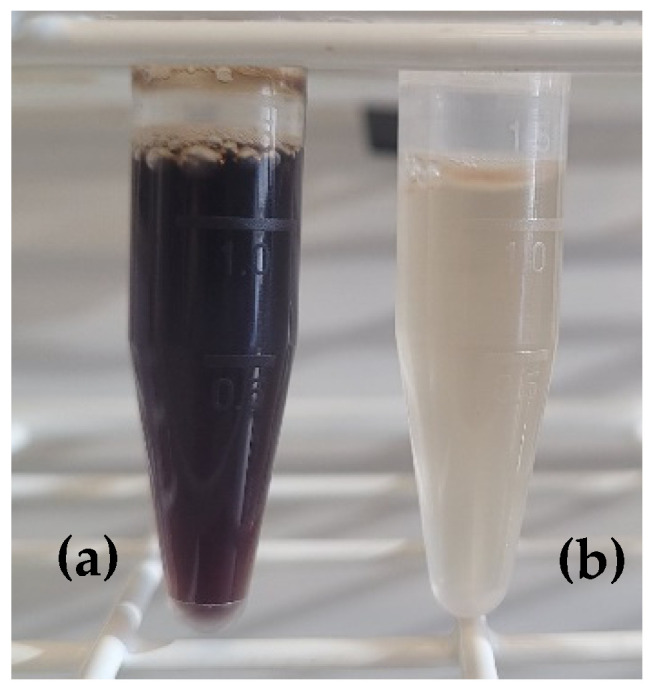
Porcine cruor raw hydrolysate (**a**) and discolored hydrolysate (**b**).

The protein content indicated a significant difference between discolored and raw hydrolysates (Student *t*-test, *p*-value = 0.02), presenting values of 80.3 ± 2.8% and 86.6 ± 1.1%, respectively. The difference obtained showed a loss of compounds that contained nitrogen, during the discoloration. Indeed, molecules of heme precipitated, which was confirmed by the different colors of the hydrolysates (Figure 2). Heme molecules contain porphyrin, a complex cyclic structure composed of 4 pyrrole rings made of nitrogen and carbons [39]. Other compounds containing nitrogen in addition to the heme, such as amino acids or/and peptides, might have precipitated. This difference in protein content suggested that the discoloration of the hydrolysate had consequences on its final composition.

### 3.2. Peptide Population and Identification 

#### 3.2.1. Mapping and Comparison of Peptides, before Discoloration

**Figure 3:**
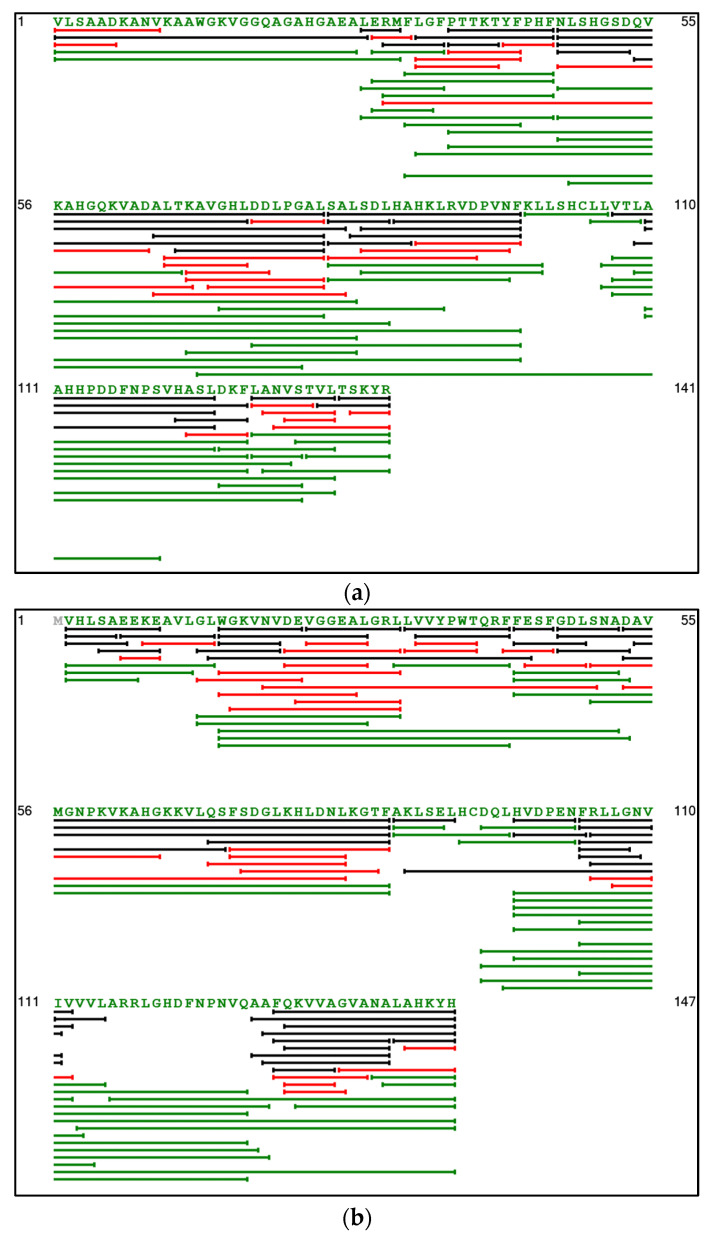
Peptide mapping comparisons of the alpha chain of porcine hemoglobin (**a**) and the beta chain of porcine hemoglobin (**b**). Green: peptides only present in the porcine cruor hydrolysate after 30 min; Red: peptides only present in the porcine hemoglobin hydrolysate after 180 min; Black: Peptides in common between hydrolysates at 30 min and 180 min.

The peptides population composing the two hydrolysates were analyzed according to their length and the number of peptide sequences, using mapping (Figure 3a,b). The peptides produced were represented for the two chains of the porcine hemoglobin and compared to a porcine hemoglobin hydrolysate of 180 min. A comparison was conducted for a better understanding of the mechanisms involved [24]. Indeed, the comparison was appropriate as the protein contained in the cruor and hydrolyzed by the pepsin was the porcine hemoglobin. Moreover, a study from Hedhili et al. demonstrated that the kinetics of cruor hydrolysis by pepsin was comparable to the purified hemoglobin. Only slight differences were observed, possibly due to the impurities present in the cruor [11]. The non-identified peptides were not represented on the mapping. The peptides are represented by lines and the length refers to the peptide size and the amino acid sequence, from the first to the last amino acid of each peptide. As expected, the peptides identified in the 30 min cruor hydrolysate had longer amino acid sequences than the 180 min porcine hemoglobin hydrolysate. Thus, the mapping illustrated a majority of higher molecular weight peptides for the 30 min hydrolysate. This variation in the peptide population can be explained by differences in the DH. Indeed, for the same pH, a DH of 8.4 ± 0.2% was obtained after 180 min [24]. With a six-time longer hydrolysis duration, the DH doubled. The evolution was not linear, and the hydrolysis slowed down between 30 and 180 min. The common peptides between the two hydrolysates were visually not as abundant as the different ones. Peptides at 30 min could be subjected to other successive cleavages, which would produce shorter and different peptides after 180 min. Nevertheless, a well-known antimicrobial peptide, named Neokyotorphin (NKT) (TSKYR), was found common in both hydrolysates. It showed that short duration of hydrolysis still allowed the production of specific low molecular masses peptides. These observations confirmed that a zipper mechanism was involved and generated intermediate and final peptides [15]. It also highlighted that NKT is a final peptide, as reported in the bovine hemoglobin hydrolysis literature [40]. The global mechanism involved in the porcine cruor peptic hydrolysis is the same as the porcine and bovine hemoglobin. The importance of the hydrolysis duration to manage the diversity of bioactivities brought by the hydrolysates was also highlighted.

#### 3.2.2. Comparison of RP-UPLC-MS/MS Raw and Discolored Profiles 

**Figure 4:**
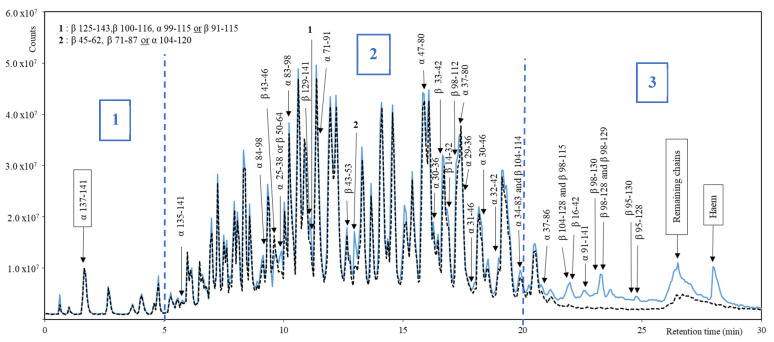
Overlay of the raw (blue plane line) and discolored (black dash line) hydrolysate spectra. Identified sequences found in the raw hydrolysate and the pellet, but absent or diminished in the discolored hydrolysate are presented on the figure with their location on the α or β chain of hemoglobin.

The profiles of the three raw hydrolysates and discolored hydrolysates repetitions were very repetitive and overlapped perfectly. One representative raw and discolored hydrolysate spectra were overlaid and divided into three parts to highlight the main trends and differences: zones 1, 2 and 3 (Figure 4). The first part (0–5 min of retention time (RT)) was similar for both studied hydrolysates’ MS spectra. The peptides contained in zone 1 were the most rapidly eluted, which indicates that they had a high polarity. Indeed, one of the main peaks identified was the Neokyotorphin peptide (α 137–141). No hydrophobic residue is carried by this peptide and its hydrophilic residues ratio is 60%. Zone 2 (5–20 min of RT) (Figure 4) represented the time interval where the most compounds eluted. The spectra presented narrow peaks and a very dense compounds population. Moreover, each peak contained one or more compounds. It was the most complex zone to study, which made the identification of peptides even more essential for a comparison of the hydrolysates. Some differences in the peaks were visually noticeable. For example, at 10 min, the raw hydrolysate presented an additional peak compared to the discolored hydrolysate, and this peak contained two peptides (α 25–38 and β 50–64). Differences also occurred inside peaks. Indeed, at 11.5 min, the two spectra presented no visual differences, but a peptide (α 71–91) was highlighted as absent in the discolored hydrolysate spectrum. Zone 3 (20–30 min of RT) (Figure 4) presented the main differences between the two hydrolysates. Indeed, fewer peaks were observed on the discolored spectrum, mainly between 21 and 25 min. The raw hydrolysate showed five peaks in this zone, which were not observed in the discolored hydrolysate. The large peak between 26 and 27 min contained the remaining chains of hemoglobin, after the 30 min of hydrolysis. Indeed, this large peak contained compounds with high molecular masses of 15,040 Da and 16,036 Da, corresponding, respectively to the α-chain and β-chain of the porcine hemoglobin (UniProtKB, P02067 and P01965). This fact was confirmed during the DH analysis which showed that no plateau was reached at final time of hydrolysis (Figure 1). The hydrolysis could be further pursued due to the presence of these remaining unhydrolyzed chains of hemoglobin. They were present in both hydrolysates, but the peak area was lower in the discolored hydrolysate. Most of these heavy compounds contained a heme group. Thus, they were affected by the precipitation and the centrifugation, applied to recover the supernatant and the pellet of the discoloration process. In the raw hydrolysate, the peak at 28 min represented the heme with a molecular mass of 616.5 Da (PubChem, CID: 53629486). No peak of heme was observed on the discolored hydrolysate spectrum, which confirmed the effectiveness of the discoloration step. Overlaying the spectra allowed to highlight some differences within peaks. The position of the main differences in zone 3 of the spectra showed some particularities. As the RT increased, meaning the gradient was increasing in acetonitrile percentage, the eluted compounds had higher molecular mass and higher hydrophobicity. Indeed, it was reported in the literature that the order of peptides elution was negatively correlated to the hydrophobicity [41]. Moreover, the highest molecular masses were observed after 19 min of RT: 7145.68 Da and 6681.44 Da at, respectively 21.2 and 19.9 min. These observations allowed to conclude that the lost peptides during the discoloration are mostly high molecular weight and hydrophobic peptides, as they were part of zone 3.

#### 3.2.3. Comparison of the Identified Peptides

A wide variety of identified peptides was obtained after 30 min of hydrolysis of the α (141 amino acids) and β (146 amino acids) porcine hemoglobin chains. The raw hydrolysate was composed of 125 identified peptides and the discolored of 123 identified peptides. A difference of 2 identified peptides was observed which suggested that a loss of peptide occurred. However, some biomolecules, including peptides, found in the hydrolysates were not identified by the matching criteria of the software. Discolored hydrolysate was composed of 295 ± 7 biomolecules whereas the raw hydrolysate of 336 ± 2 biomolecules. These differences impacted the nitrogen content of the two hydrolysates. The significant difference between contents in biomolecules of the two hydrolysates (see Section 3.1.2. protein content) was explained by a loss of peptides during the discoloration.

(a) Common peptides between raw and discolored hydrolysates

Regarding the common peptides obtained in the two hydrolysates, some known antimicrobial peptides were highlighted. Most of them are intermediate peptides of the Neokyotorphin peptide (TSKYR). It is a well-known antibacterial and antioxidant peptide [6,42]. TSKYR was found in both hydrolysates at a similar abundance (Figure 4). This peptide presented effects close to those of the butylated hydroxytoluene (BHT) used as a food additive [6]. The last peptide, at a retention time of 12 min, PTTKTYFPHF, demonstrated its bioactivity in a study on bovine hemoglobin hydrolysate [17]. This peptide was also found in the hydrolysates of this study. All the antimicrobial peptides identified with a demonstrated bioactivity are presented in Table 2.

**Figure 5:**
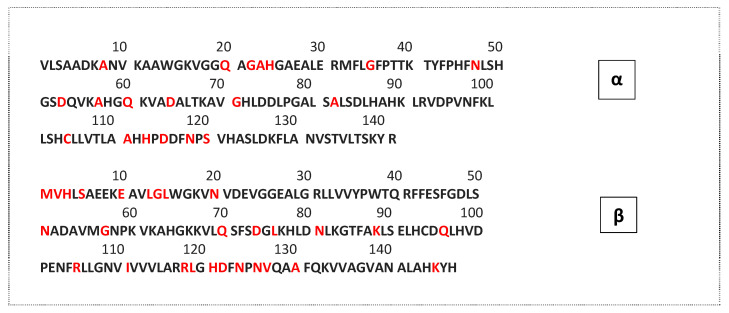
Amino acid sequence of porcine hemoglobin, α chain and β chain. Adapted from UniProtKB, Code: HP beta P02067, HP alpha P01965, HB beta P02070 and HB alpha P01966 [44]. Red amino acids are different in the bovine hemoglobin sequence.

It is essential to acknowledge that porcine and bovine hemoglobins present some differences in their amino acid sequences (Figure 5). Indeed, according to BLAST software (UniProtKB), the α chains of the two hemoglobins had 19 different amino acids, the correspondence between the two chains was 86.5%. Regarding the β chain, there were 28 different amino acids and the affinity between the two was 82.6% (UniProt Consortium, 2019). Most of the identified antimicrobial peptides were derived from bovine hemoglobin. Therefore, a comparison of the known antimicrobial peptides, reported in the literature and database, and the peptides obtained from porcine cruor hydrolysate was conducted. These peptides were qualified as potentially antimicrobial following this comparison (Table 3), since they have never been tested in a pure form. The first peptide identified: LAHKYH (β (142–147)), at the C-terminus of the β chain (Table 3 and Figure 5), was used to illustrate the procedure. In bovine hemoglobin hydrolysate, the peptide LAHRYH, has been described as an antibacterial peptide [17]. In the porcine hemoglobin, the arginine (R) is replaced by a lysine (K, position 145) amino acid (Figure 5). Both amino acids have positive electrically charged side chain. Moreover, the total hydrophobicity of the peptide was not affected by the modification of the arginine amino acid. It is known that for antimicrobial peptides, the hydrophobic character is one of the major parameters [43]. LAHKYH peptide has the potential to be an antimicrobial peptide thanks to these observations. An antimicrobial peptide database (ADP3, https://aps.unmc.edu/prediction, accessed on 1 September 2020) was also used to confirm this antimicrobial potential.

(b) Difference in peptides between raw and discolored hydrolysates

Peptide profiles of the raw hydrolysate, the discolored hydrolysate, and the discoloration pellet were compared. The aim was to detect all the differences and identify the compounds already highlighted in the comparison of spectra. Peptides identified in the raw hydrolysate and the discoloration pellet, but absent or diminished by at least 10% in the discolored hydrolysate, were emphasised. The decrease was defined as the variation of the area of the peptide in the spectra (Figure 4). A total of 41 peptides were identified as lost and recovered in the pellet during discoloration. These peptides were represented on the spectra (Figure 4) and listed with their main characteristics (Table 4). None of these peptides has already been studied for their antimicrobial activities. 

As expected, no difference was observed in zone 1 of the spectra. Concerning zone 2, amongst the 27 different peptides found, 4 peptides were absent in the discolored hydrolysate and the other peptides had a diminished area of at least 10%. The molecular masses of these peptides were mostly between 800 Da and 3000 Da. Two exceptions were given with molecular masses of 4681.4 Da for the peptide α 37–80 and 5269.7 Da, for the α 34–83. They were present in the raw hydrolysate and pellet of discoloration, but part of the absent peptides in the discolored hydrolysate, which conferred them a 100% decrease. Zone 3 contained, as expected, peptides with the highest percentage of decrease. Indeed, 12 over 14 peptides decreased by 90% and more, during discoloration. They were peptides with molecular weights between 2800 and 5800 Da (Table 4). Finally, regarding the sequences, some peptides were the intermediates of other peptides. The main examples were the lost peptides with the following location on the alpha chain: α 98–115, α 98–128, α 98–129, α 98–130 and α 95–128, α 95–130 (Table 4). 

The total hydrophobic residues, the main secondary structure, and the charge at pH 7 were presented for each identified peptide. These characteristics are usually considered when the antimicrobial potential is evaluated. Whether the peptide present antibacterial, antifungal or both activities, they usually present a positive charge (+2 to +9), a certain hydrophobicity (around 50%) and a specific structure (α helix, β sheets or a mixture) [45]. Peptides with the capacity to disrupt the membrane generally take part on the α-helical structure class [43]. Since these peptides were identified for the first time, these data helped the interpretation of these peptides’ relevance. Nevertheless, some known antimicrobial peptides, such as the Neokyotorphin with TSKYR amino acids sequence, are exceptions. Indeed, this peptide presents no hydrophobicity (Antimicrobial peptide database, ADP3, University of Nebraska Medical Center). Thus, to confirm the interest of these 38 peptides, based on these criteria, microbiological tests were carried out. The results helped to understand the real impact of these modifications in peptides profiles, on antimicrobial activities of both hydrolysates. 

### 3.3. Antimicrobial Activities

#### 3.3.1. Antibacterial Activity of the Hydrolysates 

The hydrolysates did not show any activity against the two bacterial strains tested by agar diffusion method (Table 5). Since *E. coli* and *L. ivanovii* were resistant to both hydrolysates no further antibacterial tests on other strains were carried out. The chosen duration of hydrolysis impacted the final antibacterial activities. Indeed, although potential antibacterial peptides were found in the hydrolysates (Table 2) the DH was low. Consequently, it influenced the concentration of the potential antibacterial peptides in the hydrolysate (Figure 3 and Figure 4). It is explained by the mechanism produced by the pepsin which was the “zipper” mechanism [46]. It represents reaction pathways with appearance and disappearance of peptides during the duration of hydrolysis which generates intermediate and final peptides [11]. After a duration of 30 min intermediate peptides were mainly generated with some final peptides such as NKT. UPLC MS–MS analyses revealed that 30 min was optimal to obtain the highest relative abundance of NKT compared to durations up to 180 min, which was the primary motivation to define this parameter. As porcine cruor hydrolysates are complex and the peptides are diverse, the accumulation of other antibacterial peptides (Table 2) and potential one (Table 3) was not sufficient. Indeed, for similar hydrolysis parameters porcine hemoglobin hydrolysate of 180 min showed antibacterial activity against *L. ivanovii* [24] and a porcine cruor hydrolysate during 24 h showed activity against *L. innocua* [13]. This would suggest that a longer hydrolysis duration and hence higher DH lead to the release of more low molecular mass peptides with antibacterial activities.

Those results were also different from demonstrations made with bovine hemoglobin hydrolysate. Indeed a 3% DH hydrolysate revealed antibacterial activities against *M. luteus L. innocua E. coli* and *S. enteritidis* [46]. Thus, the amino acids and cleavage sites differences [13] between the porcine and bovine hemoglobin sequences might also have an impact on the final activities. The importance of investigating each source of hemoglobin was confirmed. 

#### 3.3.2. Antifungal Activity

(a) Agar diffusion

**Figure 6:**
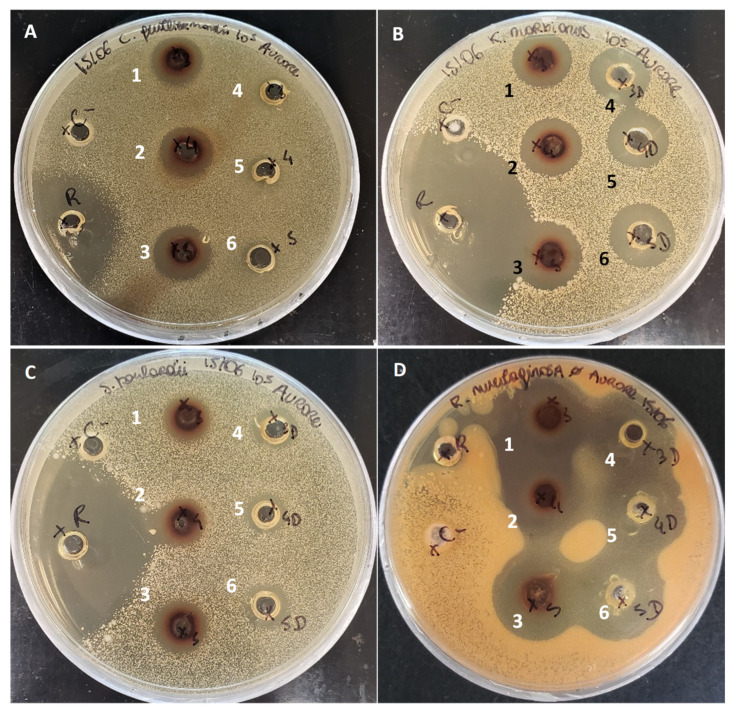
Inhibition zones for yeasts *C. guilliermondii* (**A**) *K. marxianus* (**B**) *S. boulardii* (**C**) *R. mucilaginosa* (**D**) distilled water (C –) and Reuterin (R). 1 2 and 3: Raw hydrolysate repetition 1 2 and 3; 4 5 and 6: Discolored hydrolysate repetition 1 2 and 3.

**Figure 7:**
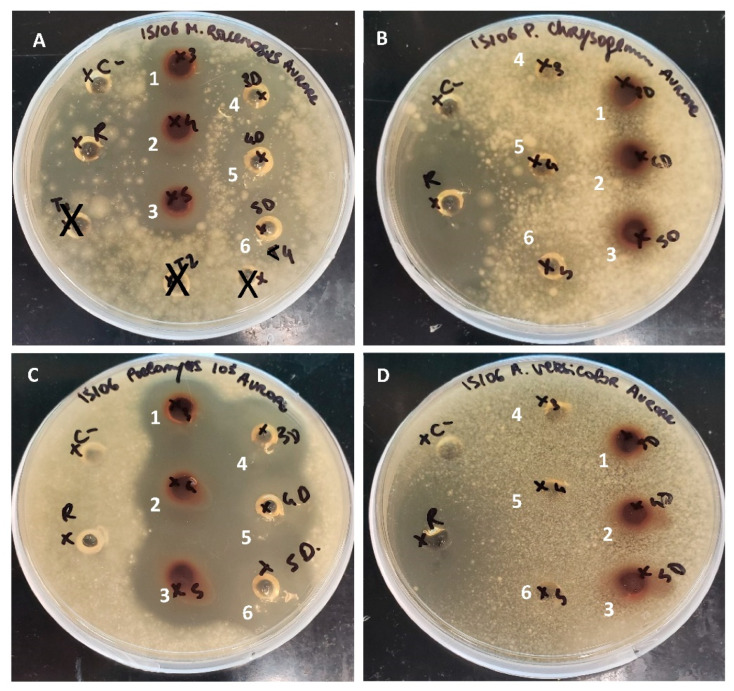
Inhibition zones for molds *M. racemosus* (**A**) *P. chrysogenum* (**B**) *Paecilomyces* spp. (**C**) *A. versicolor* (**D**) distilled water (C –) and Reuterin (R). 1 2 and 3: Raw hydrolysate repetition 1 2 and 3; 4 5 and 6: Discolored hydrolysate repetition 1 2 and 3; X: samples not relevant to the study.

Antifungal activities were demonstrated for the porcine cruor hydrolysates no matter if the discoloration step was carried out or not. Results showed inhibition against 7 strains for the raw hydrolysate, 6 strains for the discolored hydrolysate and 4 strains for the pellet produced after discoloration (Table 5). The yeast *C. guilliermondii* was significantly less sensitive (*p* < 0.05) to the discolored hydrolysate (8 ± 1 mm) compared to the raw one (15 ± 1 mm) (Figure 6). The same tendency was observed with *P. chrysogenum*. Indeed, the raw hydrolysate showed an average halo of 12 ± 1 mm compared to the discolored one which does not show any inhibition activity (Figure 7). First significant differences were observed between antifungal activity of the hydrolysates with the agar diffusion method. No activity was observed against *A. versicolor*. These tests were performed with hydrolysate samples prepared on a protein base (40 mg/mL of proteins). The protein content was measured on a nitrogen base which means that any compound with nitrogen was quantified. As the pellet was the by–product of the discoloration it contained the precipitated heme about 10.5% of its total composition. Compared to the hydrolysates the pellet tested at 40 mg/mL of proteins involved less peptides. However, this smaller amount of peptide still allowed the inhibition of four strains (Table 5 and Figure S1. Those first results demonstrated that the differences in peptide profiles impacted the antifungal activities of the hydrolysate. In addition, some of the lost peptides found in the pellet might be the source of the decreased antifungal activity for the discolored hydrolysate. Antifungal activities had already been demonstrated from bovine and porcine hemoglobin hydrolysates [10,24], but never from porcine cruor hydrolysate or on a spectrum of 8 strains.

(b) Minimal Inhibition Concentration (MIC) and Minimal Fungicidal Concentration (MFC)

Evaluation of the antifungal activities of the raw and discolored hydrolysates as well as the pellet from discoloration were performed. The MIC and MFC were determined for 8 strains of yeasts and molds. The MICs determined vary between 0.1 and 30.0 mg/mL of proteins (Table 6). The results for the different strains agreed with agar diffusion results above (Table 4). All the ratios MFC/MIC were below 4. Thus, antifungal activities observed were fungicidal activities. The yeasts *S. boulardii C. guilliermondii* and *K. marxianus* were inhibited by the raw hydrolysate at a concentration significantly lower than the discolored hydrolysate. Indeed, the raw hydrolysate was 10, 15 and 2 times more efficient against those strains, respectively. Interestingly no difference was observed with *R. mucilaginosa* which was very sensitive to the hydrolysates and the pellet. Indeed, for the raw hydrolysate this strain was 10, 8 and 7 times more sensitive compared to the three other tested yeasts. For the discolored hydrolysate *R. mucilaginosa* was 60 times more sensitive compared to *S. boulardii* and 11 times compared to *K. marxianus*. The hydrolysates were also active against molds. *M. racemosus* and *Paecilomyces* spp. were highly sensitive to the raw hydrolysate whereas *M. racemosus* was 10 times less sensitive to the discolored hydrolysate. Moreover, *P. chrysogenum* was not inhibited by the discolored hydrolysate and the pellet. Additionally, *A. versicolor* was not inhibited either by the raw or discolored hydrolysates nor the pellet. Those observations highlighted the differences in the structure and composition between genera of yeasts and molds. Especially differences in the outer cell layers generated different sensitivities [47]. Fungal cells are made of a cell wall with glycosylated proteins which are combined to more complex residues for the filamentous fungi. Then, there is the plasma membrane which is mainly composed of phospholipids, sphingolipids and sterols. The different proportions of these lipids may impact the sensitivity of the strain. The filamentous fungi are generally less sensitive due to their cell wall composition [45,48]. Different sensitivities were also observed within the tested molds this would be generated by the composition of the plasma membrane. Indeed, a study testing different sensitivity of filamentous fungi to chitosan which implies mechanisms similar to antifungal peptides showed that the presence of polyunsaturated fatty acids in the plasma membrane had a high impact on the resistant of the strain. Indeed, the membrane fluidity was easily disturbed by the binding of the molecule and caused the permeabilization of the membrane [49]. Concerning the yeasts, a study investigated the sensitivity of different strains against antimicrobial peptides and proteins from human bioliquid. They observed differences in sensitivity of tested strains and *R. mucilaginosa* was one of the most sensitive strains [50]. 

The pellet and discolored hydrolysate were both less effective than the raw hydrolysate against *S. boulardii C. guilliermondii* and *P. chrysogenum*. The pellet of discoloration was more effective than the discolored hydrolysate for the strain *K. marxianus* and *M. racemosus*. It showed interesting low concentration of inhibition against *R. mucilaginosa* and *Paecilomyces* spp. Some peptides that were present in the pellet despite their low quantity had an antifungal activity. As the activity of the discolored hydrolysate decreased peptides identified in the pellets which are also present in the raw hydrolysate could be the source of the antifungal activity. This observation showed that the difference in the composition of these two hydrolysates had a substantial impact on their antifungal activities. The results suggest also that the hydrolysis duration of porcine hemoglobin or cruor could modulate the antimicrobial activity towards antifungal or antibacterial ones.

(c) Antifungal activity of the synthesized peptides

Based on inhibition activity observed with raw and discolored hydrolysates the main lost peptides were synthesized: they represented 11 new sequences (Table 4). In order to investigate minimal antifungal sequences, which means the smallest amino acids sequence providing an antifungal activity and to explain the results, peptides found in both hydrolysates: LAHKYH NALAHKYH and HVDPEN were also synthesized and tested. It was also interesting to observe that several peptides were intermediate of others. FRLLGNVIVVV, also identified as β 104–114, is part of 6 different synthesized peptides: β 98–115, β 98–130, β 98–129, β 98–128, β 95–130 and β 98–147 (Table 4). 

Among the 14 peptides 7 peptides demonstrated inhibition against yeast and mold and peptide β 98–115 only against yeast (Table 7). The ratios MFC/MIC reported fungicide activities for the 8 effective peptides. Indeed, their ratios were all below 4. The other 6 synthesized peptides had not inhibited the strains at the highest tested concentration (1.25 mM). LAHKYH produced an antifungal activity with a MIC of 1.25 mM which was interestingly decreased by the addition of an asparagine (N) and an alanine (A) amino acid residue at the N–terminal. Indeed, the MIC of NALAHKYH was 4 times lower for *R. mucilaginosa* and 8 times for *Paecilomyces* spp. The lower the MIC is the less peptides are required to be active against the fungi. The change in sequence on amino acids increased the total hydrophobic residues from 33% to 38% [51]. This observation can explain part of the difference obtained in antimicrobial activities. It was well described that the hydrophobicity of peptides was part of the main antifungal parameters. Moreover, it was shown that increasing hydrophobicity improved the selectivity against eukaryotic membranes specially to enhance the affinity to zwitterionic lipid [52]. HVDPEN was not active against the strains tested. However, this sequence occurs in peptides β 98–115 β 95–130 and β 98–147 which were active. Those peptides also contained the segment of sequence FRLLGNVIVVV which had a MIC of 0.156 mM and 0.625 mM against the yeast and mold, respectively. As it was reported in Table 4 this peptide had the highest hydrophobicity which could explain its interesting activities. It was the most effective peptide as it had the lowest MIC and was active against both strains comparing to β 98–115. The peptide β 98–147 contained three tested sequences including two active ones. Moreover, it is formed at 78% by an α–helix which is known to promote antimicrobial activities [53]. Finally, the peptides α 71–91 and α 37–80 were two independent peptides as their sequences were not include or did not include other synthesized sequences. They are both formed at majority of α–helix structures with a lower global hydrophobicity compared to the other random coil structured peptides identified as active. 

These explanations were based on in silico results and further studies could be performed on these new antifungal peptide sequences to validate their mechanisms of action: barrel–stave pore model toroidal pore model or carpet model [52]. Agar diffusion assays were also conducted on the peptides and results are presented in Supplementary Material (Figure S2 and Table S1). The results were not as clear and precise compared to the MICs. Indeed, the good visualization of the inhibition zones was limited by the aspect of some solution containing the peptides. The method performed consisted of depositing a drop of peptide solution on the agar instead of making a well (see Section 3.3.2 a Agar diffusion). Moreover, these peptides could be tested against a larger fungi strains panel.

The synergistic effect was evaluated on the most effective antifungal peptides (β 140–147 β 104–114 β 98–115 α 71–91 and α 37–80). The interactions between each pair of peptides were evaluated. A total of 10 combinations were tested. None of the combinations has demonstrated a synergistic effect against *R. mucilaginosa* nor *Paecilomyces* spp. with the method used.

## 4. Conclusions

The peptic hydrolysis of porcine cruor produced a wide variety of peptides including known active peptides. The discolored hydrolysate by heme precipitation using acidification presented a different peptide profile. Indeed, relative abundances of 38 identified peptides were lost or diminished in the discolored hydrolysate compared to the raw hydrolysate. These peptides were also identified in the discoloration pellet. Moreover, significant differences were observed concerning antifungal activity against some strains of yeasts and molds. The discoloration pellets also demonstrated antifungal activities. The hypothesis that some of the lost peptides had a specific action on fungi was supported by these results. Thus, eight totally new sequences among the lost peptides were synthesized and demonstrated effective antifungal activities. Discoloration of the porcine cruor hydrolysate allowed the identification of new antifungal peptides and explained why discolored hydrolysate presented lower antifungal activities than the raw hydrolysate. 

Concerning antibacterial activity, 7 known sequences were identified, and 13 potentially antibacterial sequences were highlighted in both hydrolysates. It appeared from the results that these antibacterial peptides identified were not at a sufficient concentration to inhibit the strains tested. This lack of antibacterial activity was also explained by the duration of the hydrolysis of 30 min. It was concluded that the hydrolysis duration influences the final antimicrobial activity of the hydrolysate either antifungal activity with high molecular mass peptides or antibacterial activity with smaller peptides. It was the first time that such a demonstration was reported on porcine cruor. 

It appeared that the discoloration step had a major impact on the final hydrolysate’s antimicrobial activities. Both raw and discolored hydrolysates had antifungal activities which was very encouraging considering a potential future application in food preservation. However, from an application point of view in the meat industry this information is of major importance: the concentration of discolored hydrolysate if used on white meat or ham for example must be adjusted to have the same effect as the raw hydrolysate. Following these results, it appears that porcine blood has the potential to enter a circular economy by becoming the raw material of a new process: the production of antimicrobial peptides. Thus, the meat industry could produce natural preservatives from its coproducts which would represent a gain compared to the purchase of antimicrobials use of blood as low value–added product (animal meal production) and/or treatment of these wastes. In addition, the recovery yield of these peptides if the raw cruor is used as is would be close to 100% at an industrial scale. In fact, all the cruor separated from the blood by centrifugation without no other processing step would be used for hydrolysis and finally recovered to be dried or applied directly as a liquid. 

This is an innovative study demonstrating the scientific feasibility and of interest for industrials. For future perspectives membrane processes could be used to concentrate and increase these antimicrobial peptides. Indeed, two fractions: one antifungal and one antibacterial could be produced using separation processes. Consequently, the identification of the sequences of interest will allow a better separation or choice of separation processes based on their physicochemical characteristics.

## Figures and Tables

**Table 1:** Strains used for antimicrobial assays on hydrolysates and peptides.

Strain		Subculture Conditions	References	Strain Collection
Bacteria	*Escherichia coli*	TSB ^1^, 18 h, 37 °C (×2)	MC 4100	Food microbiology laboratory (LMA), Laval University, Québec (QC) Canada
	*Listeria ivanovii*	TSB, 18 h, 30 °C (×2)	HPB 28	
Filamentous Molds	*Mucor racemosus*	PDA ^2^, 7 days, 25 °C (×2)	LMA-722	
	*Penicillium chrysogenum*		LMA-212	
	*Aspergillus versicolor*		LMA-370	
	*Paecilomyces* spp.		5332-9a	Denis Roy, Laval University
Yeasts	*Rhodotorula mucilaginosa*	PDB ^3^, 48 h, 25 °C (×2–3)	27,173	General Mills Yoplait, France
	*Saccharomyces boulardii*		27,169	
	*Kluyveromyces marxianus*		27,175	
	*Candida guilliermondii*		27,168	

^1^ TSB: Tryptic Soy Broth (BD-Difco, Sparks, MD, USA); ^2^ PDA: Potato Dextrose Agar (BD-Difco, Sparks, MD, USA; ^3^ PDB: Potato Dextrose Broth (BD-Difco, Sparks, MD, USA). (×_): number of subcultures.

**Table 2:** Antimicrobial peptides recovered from the porcine cruor hydrolysate.

Retention Time (min)	Molecular Mass (Da)	Chain	Location	Sequence *
1.64	653.35	α	137–141	TSKYR
5.97	865.50	α	135–141	VLTSKYR
6.54	966.55	α	134–141	TVLTSKYR
6.67	1053.58	α	133–141	STVLTSKYR
8.45	1337.73	α	130–141	ANVSTVLTSKYR
10.36	1450.81	α	129–141	LANVSTVLTSKYR
12.01	1237.61	α	37–46	PTTKTYFPHF

* According to results reported in the literature from bovine hemoglobin [13].

**Table 3:** Potential antimicrobial peptides from the porcine cruor hydrolysate.

Retention Time (min)	Molecular Mass (Da)	Chain	Location	Sequence *
4.57	767.41	β	142–147	LAH**K**YH
8.01	812.46	α	99–105	KLLSH**C**L
9.15	2633.37	α	1–28	VLSAADK**A**NVKAAWGKVGG**Q**A**GAH**GAEA
10.86	2746.44	α	1–29	VLSAADKANVKAAWGKVGG**Q**A**GAH**GAEAL
10.93	925.54	α	99–106	KLLSH**C**LL
11.37	2417.17	α	107–128	VTLA**A**H**H**P**D**DF**N**P**S**VHASLDKF
12.14	3162.64	α	1–32	VLSAADK**A**NVKAAWGKVGG**Q**A**GAH**GAEALERM
16.46	1554.78	α	34–46	L**G**FPTTKTYFPHF
19.13	1701.85	α	33–46	FL**G**FPTTKTYFPHF
19.89	6681.47	α	37–98	PTTKTYFPHF**N**LSHGS**D**QVK**A**HG**Q**KVA**D**ALTKAV**G**HLDDLPGALS**A**LSDLHAHKLRVDPVNF
20.60	3214.64	α	107–136	VTLA**A**H**H**P**D**DF**N**P**S**VHASLDKFLANVSTVL
21.29	7145.68	α	33–98	FL**G**FPTTKTYFPHF**N**LSHGS**D**QVK**A**HG**Q**KVA**D**ALTKAVGHLDDLPGALS**A**LSDLHAHKLRVDPVNF

* Amino acids in bold: Differences with bovine hemoglobin sequences, same location on the chain.

**Table 4:** Peptides absent or diminished in the discolored porcine cruor hydrolysates.

Retention Time (min)	Molecular Mass (Da)	Chain	Location	Sequence	Total Hydrophobic Residues (%) ^2^	Structure (%) ^3^	Charge at pH 7 ^2^	Decrease (%) *
5.97	865.501	α	135–141	VLTSKYR	29	RC 100	+2.0	10.1
9.34	1746.912	α	84–98	SDLHAHKLRVDPVNF	47	RC 60	+0.2	12.0
9.60	528.221	β	43–46	FESF	50	RC 100	−1.0	10.8
9.91	1537.760	α	25–38	GAEALERMFLGFPT ^1^	50	AH 30	−1.0	38.0
	1537.767	β	50–64	SNADAVMGNPKVKAH ^1^	40	RC 80	+1.3	
10.23	1859.997	α	83–98	LSDLHAHKLRVDPVNF	50	RC 81	+0.2	22.5
10.86	1244.689	β	129–141	AAFQKVVAGVANA	69	AH 70	+1.0	100.0
11.10	1867.032	β	125–143	PNVQAAFQKVVAGVANALA ^1^	63	AH 84	+1.0	38.0
	1867.057	β	100–116	DPENFRLLGNVIVVVLA ^1^	58	AH 47	−1.0	
	1867.014	α	99–115	KLLSHCLLVTLAAHHPD ^1^	52	RC 70	+0.8	
	2870.511	β	91–115	ELHCDQLHVDPENFRLLGNVIVVVL ^1^	52	RC 52	−2.5	45.0
**11.50**	**2179.146**	**α**	**71–91**	**GHLDDLPGALSALSDLHAHKL**	**42**	**AH 57**	**−1.3**	**35.0**
12.20	1200.493	β	43–53	FESFGDLSNAD	36	RC 100	−3.0	100.0
12.90	1848.883	α	104–120	CLLVTLAAHHPDDFNPS ^1^	47	RC 77	−1.5	46.0
	1848.904	β	45–62	SFGDLSNADAVMGNPKVK ^1^	38	RC 100	0	
	1848.937	β	71–87	SFSDGLKHLDNLKGTFA ^1^	35	AH 53	+0.3	
15.89	3461.789	α	47–80	NLSHGSDQVKAHGQKVADALTKAVGHLDDLPGAL	41	AH 68	−0.7	16.6
16.27	898.436	α	30–36	ERMFLGF	57	AH 43	0	17.5
16.65	1307.706	β	33–42	LVVYPWTQRF	60	AH 60	+1.0	11.6
16.84	1968.038	β	14–32	GLWGKVNVDEVGGEALGRL	42	AH 79	−1.0	49.9
17.16	1720.924	β	98–112	HVDPENFRLLGNVIV	46	RC 40	−0.8	25.0
**17.35**	**4681.402**	**α**	**37–80**	**PTTKTYFPHFNLSHGSDQVKAHGQKVADALTKAVGHLDDLPGAL**	**34**	**AH 52**	**+1.0**	**100.0**
17.60	1011.521	α	29–36	LERMFLGF	63	RC 63	0	20.4
17.99	1988.996	α	31–46	RMFLGFPTTKTYFPHF	50	RC 88	+2.1	22.6
18.24	2118.036	α	30–46	ERMFLGFPTTKTYFPHF	47	RC 100	+1.1	19.8
19.00	1420.785	β	32–42	LLVVYPWTQRF	64	RC 55	+1.0	47.3
19.96	5269.722	α	34–83	LGFPTTKTYFPHFNLSHGSDQVKAHGQKVADALTKAVGHLDDLPGALSAL	38	AH 60	+1.0	100.0
**20.02**	**1227.766**	**β**	**104–114**	**FRLLGNVIVVV**	**72**	**RC 46**	**+1.0**	**46.0**
20.78	5267.677	α	37–86	PTTKTYFPHFNLSHGSDQVKAHGQKVADALTKAVGHLDDLPGALSALSDL	36	AH 60	0	100.0
21.87	2845.597	β	104–128	FRLLGNVIVVVLARRLGHDFNPNVQ	52	RC 50	+2.3	100.0
**21.99**	**2032.146**	**β**	**98–115**	**HVDPENFRLLGNVIVVVL**	**61**	**RC 72**	**−1.0**	**63.9**
22.12	3087.608	β	16–42	WGKVNVDEVGGEALGRLLVVYPWTQRF	44	RC 60	0	100.0
22.57	5698.005	α	91–141	LRVDPVNFKLLSHCLLVTLAAHHPDDFNPSVHASLDKFLANVSTVLTSKYR	45	AH 60	+2.0	100.0
**23.2**	**3678.966**	**β**	**98–130**	**HVDPENFRLLGNVIVVVLARRLGHDFNPNVQAA**	**48**	**AH 52**	**+0.5**	**92.0**
**23.26**	**3536.894**	**β**	**98–128**	**HVDPENFRLLGNVIVVVLARRLGHDFNPNVQ**	**45**	**RC 61**	**+0.5**	**90.0**
**23.33**	**3607.928**	**β**	**98–129**	**HVDPENFRLLGNVIVVVLARRLGHDFNPNVQA**	**53**	**RC 72**	**+0.2**	**100.0**
**24.73**	**4035.127**	**β**	**95–130**	**DQLHVDPENFRLLGNVIVVVLARRLGHDFNPNVQAA**	**47**	**RC 56**	**−0.5**	**100.0**
24.79	3893.056	β	95–128	DQLHVDPENFRLLGNVIVVVLARRLGHDFNPNVQ	44	AH 50	−0.5	100.0
**26.38**	**4270.106**	**β**	**16–53**	**WGKVNVDEVGGEALGRLLVVYPWTQRFFESFGDLSNAD**	**42**	**RC 53**	**−3.0**	**100.0**
**26.45**	**4155.071**	**β**	**16–52**	**WGKVNVDEVGGEALGRLLVVYPWTQRFFESFGDLSNA**	**43**	**RC 57**	**−2.0**	**100.0**
**26.51**	**5512.961**	**β**	**98–147**	**HVDPENFRLLGNVIVVVLARRLGHDFNPNVQAAFQKVVAGVANALAHKYH**	**50**	**AH 78**	**+3.0**	**100.0**

^1^ Identification using ExPASy FindPept tool. ^2^ Antimicrobial peptide database (https://aps.unmc.edu/prediction, accessed on 1 September 2020). ^3^ (https://npsa–prabi.ibcp.fr/, accessed on 1 June 2021) Prediction of secondary structure using a consensus prediction method (MLRC DSC and PHD) RC: Random Coil AH: Alpha Helix (the main structure formation was represented). * Variation of the peak area of the peptide between the spectra expressed in %. Sequences in bold are the synthesized peptides.

**Table 5:** Antimicrobial activity of porcine cruor hydrolysates.

	Strains	Strain No.	Raw Hydrolysate	Discolored Hydrolysate	Pellet from Discoloration
Bacteria	*E. coli*	MC4100	−	−	n/a
	*L. ivanovii*	HPB28	−	−	n/a
Yeasts	*R. mucilaginosa*	27173	+++	+++	++
	*C. guilliermondii*	27168	++	+	−
	*S. boulardii*	27169	+	+	−
	*K. marxianus*	27175	++	++	+
Molds	*M. racemosus*	LMA–722	++	++	+
	*P. chrysogenum*	LMA–212	+	−	−
	*A. versicolor*	LMA–370	−	−	−
	*Paecilomyces* spp.	5332–9a	+++	+++	++

Inhibition zones: +++: ≥20 mm; ++: 15–20 mm; +: <15 mm and −: no activity. n/a: not applicable.

**Table 6:** Minimal Inhibitory Concentration (MIC) and Minimal Fungicidal Concentration (MFC) of raw and discolored porcine cruor hydrolysates on different strains of yeasts and molds.

Strain	mg/mL	Hydrolysate		
		Raw	Discolored	Pellet
*S. boulardii*	MIC	2.9 ± 0.0 ^a^	30.0 ± 0.0 ^b^	>40± 0.0 ^c^
	MFC	5.9 ± 0.6 ^a^	40.0 ± 0.0 ^b^	n.d.
	MFC/MIC Ratio	2.0	1.3	n.d.
*C. guilliermondii*	MIC	2.5 ± 0.0^a^	>40.0 ± 0.0 ^b^	>40.0 ± 0.0 ^b^
	MFC	3.8 ± 0.0	n.d.	n.d.
	MFC/MIC Ratio	1.5	n.d.	n.d.
*K. marxianus*	MIC	2.1 ± 0.0 ^a^	5.8 ± 0.0 ^c^	2.5 ± 0.0 ^b^
	MFC	5.6 ± 1.3 ^a^	7.8 ± 2.6 ^b^	10.0 ± 0.0 ^c^
	MFC/MIC Ratio	2.7	1.3	4.0
*R. mucilaginosa*	MIC	0.3 ± 0.0 ^a^	0.5 ± 0.2 ^a^	0.8 ± 0.0 ^b^
	MFC	0.3 ± 0.0 ^a^	0.5 ± 0.2 ^a^	1.3 ± 0.0 ^b^
	MFC/MIC Ratio	1.0	1.0	1.5
*M. racemosus*	MIC	0.7 ± 0.2 ^a^	8.9 ± 2.9 ^c^	4.7 ± 0.5 ^b^
	MFC	0.8 ± 0.4 ^a^	8.9 ± 2.9 ^c^	5.0 ± 0.0 ^b^
	MFC/MIC Ratio	1.1	1.0	1.1
*Paecilomyces* spp.	MIC	0.1 ± 0.0 ^a^	0.1 ± 0.0 ^a^	0.7 ± 0.1 ^b^
	MFC	0.1 ± 0.0 ^a^	0.1 ± 0.0 ^a^	0.7 ± 0.1 ^b^
	MFC/MIC Ratio	1.0	1.0	1.0
*P. chrysogenum*	MIC	12.5 ± 0.0 ^a^	>40.0 ± 0.0 ^b^	>40.0 ± 0.0 ^b^
	MFC	12.5 ± 0.0	n.d.	n.d.
	MFC/MIC Ratio	1.0	n.d.	n.d.
*A. versicolor*	MIC	>40.0 ± 0.0 ^a^	>40.0 ± 0.0 ^a^	>40.0 ± 0.0 ^a^
	MFC	n.d.	n.d.	n.d.
	MFC/MIC Ratio	n.d.	n.d.	n.d.

Values with a common letter are not significantly different according to the Kruskal–Wallis one way analysis of variance on ranks followed by a multiple test comparison: Tukey test (*p* < 0.05). Data >40.0 ± 0.0 mg/mL are not active at the tested concentration. n.d.: not determined.

**Table 7:** Minimal Inhibitory Concentration (MIC) and Minimal Fungicidal Concentration (MFC) of the synthesized peptides for a yeast and a mold indicator strains.

Sequence (Amino Acids)		*R. mucilaginosa* (mM)	*Paecilomyces* spp. (mM)
LAHKYH	MIC	1.3 ± 0.0	1.3 ± 0.0
	MFC	1.3 ± 0.0	1.3 ± 0.0
	MFC/MIC	1.0	1.0
NALAHKYH	MIC	0.3 ± 0.0	0.2 ± 0.0
	MFC	0.3 ± 0.0	0.3 ± 0.0
	MFC/MIC	1.0	2.0
FRLLGNVIVVV	MIC	0.2 ± 0.0	0.6 ± 0.0
	MFC	0.3 ± 0.0	0.6 ± 0.0
	MFC/MIC	2.0	1.0
HVDPENFRLLGNVIVVVL	MIC	0.10 ± 0.05	>1.3 ± 0.0
	MFC	0.3 ± 0.1	n.d.
	MFC/MIC	2.0	n.d.
DQLHVDPENFRLLGNVIVVVLARRLGHDFNPNVQAA	MIC	1.3 ± 0.0	1.3 ± 0.0
	MFC	1.3 ± 0.0	1.3 ± 0.0
	MFC/MIC	1.0	1.0
GHLDDLPGALSALSDLHAHKL	MIC	0.6 ± 0.0	1.3 ± 0.0
	MFC	0.6 ± 0.0	1.7 ± 0.7
	MFC/MIC	1.0	0.8
PTTKTYFPHFNLSHGSDQVKAHGQKVADALTKAVGHLDDLPGAL	MIC	0.3 ± 0.0	0.05 ± 0.02
	MFC	0.3 ± 0.0	0.07 ± 0.00
	MFC/MIC	1.0	1.0
HVDPENFRLLGNVIVVVLARRLGHDFNPNVQAAFQKVVAGVANALAHKYH	MIC	0.8 ± 0.4	0.8 ± 0.4
	MFC	0.8 ± 0.4	1.3 ± 0.0
	MFC/MIC	1.0	1.3

## Data Availability

The data used to support the findings of this study can be made available by the corresponding author upon request.

## Supplementary Materials

The following supporting information can be downloaded at: https://www.mdpi.com/article/10.3390/foods11244035/s1. Figure S1: Agar diffusion test of the porcine cruor hydrolysate discoloration pellets; Figure S2: Agar diffusion test of the synthesized peptides, inhibition zones for *R. mucilaginosa* and *Paecilomyces* spp.; Table S1. Identification of peptide sequence according to their number.

## References

[1] Canadian Pork Council Foreign Trade. https://www.cpc-ccp.com/foreign-trade.

[2] MAPAQ Élevage de Porc. https://www.mapaq.gouv.qc.ca/fr/Productions/Production/Pages/Porc.aspx.

[3] (2019). Government of Ontario Chapitre 7-Élimination des Déchets de Production de Viande.

[4] Agriculture and Agri-Food Canada Porc. https://agriculture.canada.ca/fr/secteurs-agricoles-du-canada/production-animale/information-marche-viandes-rouges/porc.

[5] (2014). Government of New-Brunswick Lignes Directrices Régissant l’élimination Des Déchets d’abattoirs et Des Carcasses.

[6] Przybylski R., Firdaous L., Châtaigné G., Dhulster P., Nedjar N. (2016). Production of an antimicrobial peptide derived from slaughterhouse by-product and its potential application on meat as preservative. Food Chem..

[7] Román S., Sánchez-Siles L.M., Siegrist M. (2017). The importance of food naturalness for consumers: Results of a systematic review. Trends Food Sci. Technol..

[8] Zhou G., Xu X., Liu Y. (2010). Preservation technologies for fresh meat—A review. Meat Sci..

[9] Leyva Salas M., Mounier J., Valence F., Coton M., Thierry A., Coton E. (2017). Antifungal Microbial Agents for Food Biopreservation—A Review. Microorganisms.

[10] Abou-Diab M., Thibodeau J., Deracinois B., Flahaut C., Fliss I., Dhulster P., Bazinet L., Nedjar N. (2020). Bovine Hemoglobin Enzymatic Hydrolysis by a New Eco-Efficient Process-Part II: Production of Bioactive Peptides. Membranes.

[11] Hedhili K., Dimitrov K., Vauchel P., Sila A., Chataigné G., Dhulster P., Nedjar N. (2015). Valorization of cruor slaughterhouse by-product by enzymatic hydrolysis for the production of antibacterial peptides: Focus on α 1–32 family peptides mechanism and kinetics modeling. Bioprocess Biosyst. Eng..

[12] Ivanov V.T., Karelin A.A., Philippova M.M., Nazimov I.V., Pletnev V.Z. (1997). Hemoglobin as a Source of Endogenous Bioactive Peptides: The Concept of Tissue-Specific Peptide Pool. Pept. Sci..

[13] Nedjar-Arroume N., Zouari O., Przybylski R., Hannioui M., Sion L., Dhulster P. (2020). High Added-Value Co-Product: The Porcine Cruor is an Attractive Source of Active Peptides. J. Nutr. Health Food Sci..

[14] Wei J.-T., Chiang B.-H. (2008). Bioactive peptide production by hydrolysis of porcine blood proteins in a continuous enzymatic membrane reactor. J. Sci. Food Agric..

[15] Adje E.Y., Balti R., Kouach M., Dhulster P., Guillochon D., Nedjar-Arroume N. (2011). Obtaining antimicrobial peptides by controlled peptic hydrolysis of bovine hemoglobin. Int. J. Biol. Macromol..

[16] Chang C.-Y., Wu K.-C., Chiang S.-H. (2007). Antioxidant properties and protein compositions of porcine haemoglobin hydrolysates. Food Chem..

[17] Nedjar-Arroume N., Dubois-Delval V., Adje E.Y., Traisnel J., Krier F., Mary P., Kouach M., Briand G., Guillochon D. (2008). Bovine hemoglobin: An attractive source of antibacterial peptides. Peptides.

[18] Fogaça A.C., da Silva P.I., Miranda M.T.M., Bianchi A.G., Miranda A., Ribolla P.E., Daffre S. (1999). Antimicrobial Activity of a Bovine Hemoglobin Fragment in the Tick Boophilus microplus. J. Biol. Chem..

[19] Pereira A., Gomide L., Cecon P., Fontes E., Fontes P., Ramos E., Vidigal J. (2014). Evaluation of mortadella formulated with carbon monoxide-treated porcine blood. Meat Sci..

[20] Toldrà M., Parés D., Saguer E., Carretero C. (2011). Hemoglobin hydrolysates from porcine blood obtained through enzymatic hydrolysis assisted by high hydrostatic pressure processing. Innov. Food Sci. Emerg. Technol..

[21] Przybylski R., Bazinet L., Firdaous L., Kouach M., Goossens J.-F., Dhulster P., Nedjar N. (2019). Harnessing slaughterhouse by-products: From wastes to high-added value natural food preservative. Food Chem..

[22] Crosby W.H., Munn J.I., Furth F.W. (1954). Standardizing a method for clinical hemoglobinometry. United States Armed Forces Med. J..

[23] Vanhoute M., Firdaous L., Bazinet L., Froidevaux R., Lecouturier D., Guillochon D., Dhulster P. (2010). Effect of haem on the fractionation of bovine haemoglobin peptic hydrolysate by electrodialysis with ultrafiltration membranes. J. Membr. Sci..

[24] Sanchez-Reinoso Z., Cournoyer A., Thibodeau J., Ben Said L., Fliss I., Bazinet L., Mikhaylin S. (2021). Effect of pH on the Antimicrobial Activity and Peptide Population of Pepsin Hydrolysates Derived from Bovine and Porcine Hemoglobins. ACS Food Sci. Technol..

[25] Przybylski R., Bazinet L., Firdaous L., Kouach M., Goossens J.-F., Dhulster P., Nedjar-Arroume N. (2020). Electroseparation of Slaughterhouse By-Product: Antimicrobial Peptide Enrichment by pH Modification. Membranes.

[26] Church F.C., Swaisgood H.E., Porter D.H., Catignani G.L. (1983). Spectrophotometric Assay Using o-Phthaldialdehyde for Determination of Proteolysis in Milk and Isolated Milk Proteins. J. Dairy Sci..

[27] Adler-Nissen J. (1986). Enzymic Hydrolysis of Food Proteins.

[28] Navarrete del Toro M.A., García-Carreño F.L. (2002). Evaluation of the Progress of Protein Hydrolysis. Curr. Protoc. Food Anal. Chem..

[29] Wang G., Li X., Wang Z. (2016). APD3: The antimicrobial peptide database as a tool for research and education. Nucleic Acids Res..

[30] Fields G.B., Noble R.L. (2009). Solid phase peptide synthesis utilizing 9-fluorenylmethoxycarbonyl amino acids. Int. J. Pept. Protein Res..

[31] Sb Peptide—Synthesis & Engineering Peptide Solubility Guidelines. https://www.sb-peptide.com/support/solubility/.

[32] Vimont A., Fernandez B., Ahmed G., Fortin H.-P., Fliss I. (2018). Quantitative antifungal activity of reuterin against food isolates of yeasts and moulds and its potential application in yogurt. Int. J. Food Microbiol..

[33] Froidevaux R., Krier F., Nedjar-Arroume N., Vercaigne-Marko D., Kosciarz E., Ruckebusch C., Dhulster P., Guillochon D. (2001). Antibacterial activity of a pepsin-derived bovine hemoglobin fragment. FEBS Lett..

[34] Pfaller M.A., Sheehan D.J., Rex J.H. (2004). Determination of Fungicidal Activities against Yeasts and Molds: Lessons Learned from Bactericidal Testing and the Need for Standardization. Clin. Microbiol. Rev..

[35] Hsieh M.H., Yu C.M., Yu V.L., Chow J.W. (1993). Synergy assessed by checkerboard a critical analysis. Diagn. Microbiol. Infect. Dis..

[36] Deng H., Zheng J., Zhang F., Wang Y., Kan J. (2014). Isolation of angiotensin I-converting enzyme inhibitor from pepsin hydrolysate of porcine hemoglobin. Eur. Food Res. Technol..

[37] Dubois V., Nedjar-Arroume N., Guillochon D. (2005). Influence of pH on the Appearance of Active Peptides in the Course of Peptic Hydrolysis of Bovine Haemoglobin. Prep. Biochem. Biotechnol..

[38] Abou-Diab M., Thibodeau J., Deracinois B., Flahaut C., Fliss I., Dhulster P., Nedjar N., Bazinet L. (2020). Bovine Hemoglobin Enzymatic Hydrolysis by a New Ecoefficient Process—Part I: Feasibility of Electrodialysis with Bipolar Membrane and Production of Neokyotophin (α137-141). Membranes.

[39] Koshland D.E., Haurowitz F. (2022). “Protein”; Encyclopedia Britannica. https://www.britannica.com/science/protein.

[40] Lignot B., Froidevaux R. (1999). Solvent Effect on Kinetics of Appearance of Neokyotorphin, VV-Haemorphin-4 and a Bradykinin-Potentiating Peptide in the Course of Peptic Hydrolysis of Bovine Haemoglobin. Biotechnol. Appl. Biochem..

[41] Žuvela P., Liu J.J., Wong M.W., Bączek T. (2020). Prediction of Chromatographic Elution Order of Analytical Mixtures Based on Quantitative Structure-Retention Relationships and Multi-Objective Optimization. Molecules.

[42] Catiau L., Traisnel J., Delval-Dubois V., Chihib N.-E., Guillochon D., Nedjar-Arroume N. (2011). Minimal antimicrobial peptidic sequence from hemoglobin alpha-chain: KYR. Peptides.

[43] Powers J.-P.S., Hancock R.E. (2003). The relationship between peptide structure and antibacterial activity. Peptides.

[44] UniProt Consortium HBB—Hemoglobin Subunit Beta—Sus Scrofa (Pig)—HBB Gene & Protein. https://www.uniprot.org/uniprot/P02067.

[45] van der Weerden N.L., Bleackley M.R., Anderson M.A. (2013). Properties and mechanisms of action of naturally occurring antifungal peptides. Cell. Mol. Life Sci..

[46] Nedjar-Arroume N., Dubois-Delval V., Miloudi K., Daoud R., Krier F., Kouach M., Briand G., Guillochon D. (2006). Isolation and characterization of four antibacterial peptides from bovine hemoglobin. Peptides.

[47] Russell A.D. (2003). Similarities and differences in the responses of microorganisms to biocides. J. Antimicrob. Chemother..

[48] Matsuzaki K. (1999). Why and how are peptide–lipid interactions utilized for self-defense? Magainins and tachyplesins as archetypes. Biochim. Biophys. Acta (BBA)-Biomembr..

[49] Palma-Guerrero J., Lopez-Jimenez J.A., Pérez-Berná A.J., Huang I.-C., Jansson H.-B., Salinas J., Villalaín J., Read N.D., Lopez-Llorca L.V. (2010). Membrane fluidity determines sensitivity of filamentous fungi to chitosan. Mol. Microbiol..

[50] Arzumanian V., Erofeeva T., Zhigalkina P., Ixanova A., Svitich O. (2019). Activity of antimicrobial peptide fractions of human serum and saliva against clinically important yeasts. Curr. Top. Pept. Protein Res..

[51] University of Nebraska Antimicrobial Peptide Calculator and Predictor. https://aps.unmc.edu/prediction/predict.

[52] Soltani S., Keymanesh K., Sardari S. (2007). In silico analysis of Antifungal Peptides: Determining the Lead Template Sequence of Potent antifungal peptides. Expert Opin. Drug Discov..

[53] Shai Y., Oren Z. (2001). From “carpet” mechanism to de-novo designed diastereomeric cell-selective antimicrobial peptides. Peptides.

