# Supplementary material for: How Discoloration of Porcine Cruor Hydrolysate Allowed the Identification of New Antifungal Peptides
Source: Foods. 2022 Dec 14;11(24):4035. doi: 10.3390/foods11244035 (PMC9778238; doi:10.3390/foods11244035)
Supplement: Supplementary file 1 [file foods-11-04035-s001.zip › foods-2029222-supplementary.pdf]

Supplementary Material S1

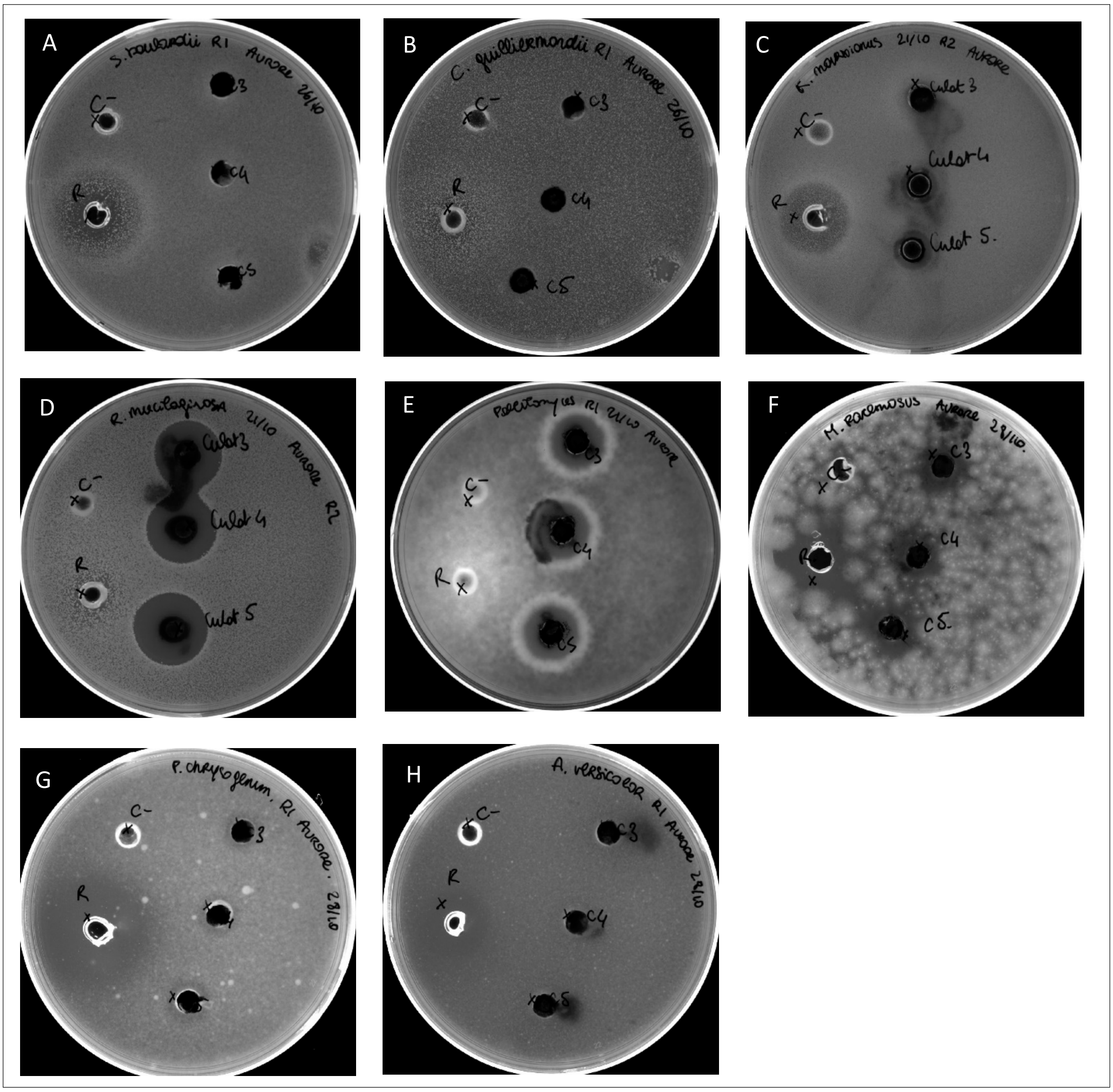

Figure S1. Agar diffusion test of the porcine cruor hydrolysate discoloration pellets. C3 (or C4, C5) or Culot 3 (or 4, 5): a repetition of a pellet of discoloration; *S. boulardii* (A), *C. guilliermondii* (B), *K. marxianus* (C), *R. mucilaginosa* (D), *Paecilomyces* spp. (E), *M. racemosus* (F), *P. chrysogenum* (G), *A. versicolor* (H), distilled water (C -) and Reuterin (R).

Supplementary Material S2

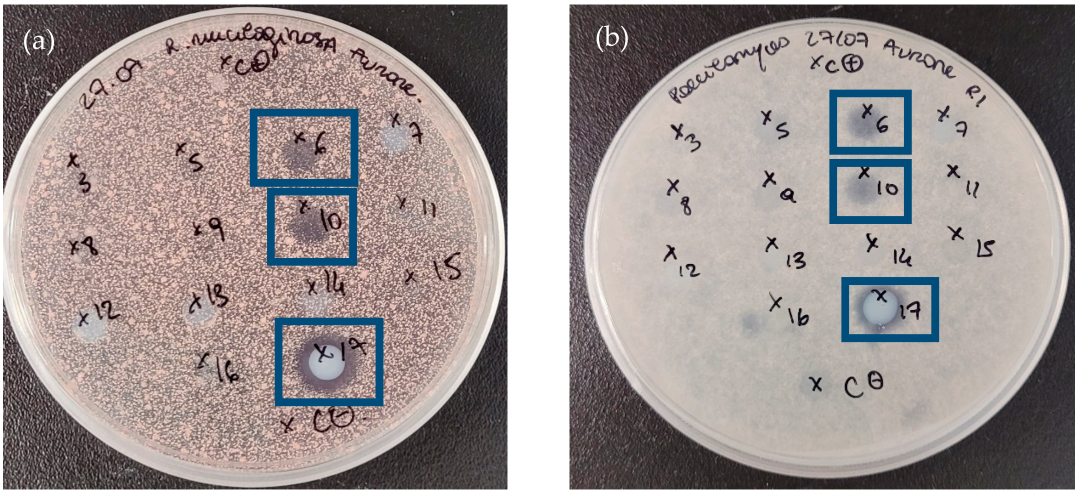

Figure S2. Agar diffusion test of the synthesized peptides, inhibition zones for *R. mucilaginosa* (a) and *Paecilomyces* spp. (b). Identification of the peptides are presented Table S1.

Table S1. Identification of peptide sequence according to their number (Figure S2)

Peptides	Sequence (Amino acids)
3	FRLLGNVIVVV
5	GHLDDLPGALSALSDLHAHKL
6	PTTKTYFPHFNLSHGSDQVKAHGQKVADALTKAVGHLDDLPGAL
7	HVDPENFRLLGNVIVVVLARRLGHDFNPNVQA
8	LAHKYH
9	HVDPEN
10	NALAHKYH
11	HVDPENFRLLGNVIVVVL
12	HVDPENFRLLGNVIVVVLARRLGHDFNPNVQAA
13	HVDPENFRLLGNVIVVVLARRLGHDFNPNVQ
14	DQLHVDPENFRLLGNVIVVVLARRLGHDFNPNVQAA
15	WGKVNVDEVGGEALGRLLVVYPWTQRFFESFGDLSNAD
16	WGKVNVDEVGGEALGRLLVVYPWTQRFFESFGDLSNA
17	HVDPENFRLLGNVIVVVLARRLGHDFNPNVQAAFQKVVAGVANALAHKYH
